# Ramadan intermittent fasting is associated with improved anticoagulant activity among healthy people: a case–control study

**DOI:** 10.1038/s41598-024-64582-8

**Published:** 2024-06-15

**Authors:** Abeer Khalid Al-Ghumlas

**Affiliations:** The Coagulation Research Laboratory, Physiology Department, College of Medicine and King Saud University Medical City, King Saud University, 11461 Riyadh, Saudi Arabia

**Keywords:** Physiology, Cardiology, Medical research

## Abstract

Data on the pathophysiological mechanisms of hemostatic alterations in the thrombotic events that occur during Ramadan intermittent fasting (RIF), particularly in the natural coagulation inhibitors, are very limited. Thus, our objective was to evaluate the effect of RIF on the natural anticoagulants level, antithrombin, protein C, and total and free protein S (PS) in healthy participants. Participants were divided into two groups. Group I consisted of 29 healthy fasting participants whose blood samples were taken after 20 days of fasting. Group II included 40 healthy non-fasting participants whose blood samples were taken 2–4 weeks before the month of Ramadan. Coagulation screening tests including prothrombin time (PT), activated partial thromboplastin time (APTT) and plasma fibrinogen level, natural anticoagulants; antithrombin, protein C, free and total PS and C4 binding protein (C4BP) levels were evaluated in the two groups. High levels of total and free PS without change in antithrombin, protein C, and C4BP levels were noted in the fasting group as compared with non-fasting ones (p < 0.05). PT and APTT showed no difference between the two groups. However, the fibrinogen level was higher in the fasting group. In conclusion, RIF was found to be associated with improved anticoagulant activity in healthy participants, which may provide temporal physiological protection against the development of thrombosis in healthy fasting people.

## Introduction

Ramadan is the 9th lunar month of the Islamic calendar during which Muslims abstain from eating food and drink from dawn to sunset. In recent years, the radical change in the lifestyle associated with this intermittent fasting (IF) and its potential effect on body function both in health and diseases has been the subject of numerous studies that documented biochemical^1–4^, hematological^5^, and hormonal^6,7^ changes. Besides, RIF has an improving effect on cardiometabolic risk factors, providing transient protection against cardiovascular disease among healthy people^8,9^. However, a significant association has also been reported between Ramadan fasting and the occurrence of cerebrovascular thrombosis^10,11^ as well as the risk of sinus venous thrombosis, retinal vein occlusion^12,13^, and acute coronary syndrome^14–16^.

These studies raised a strong possibility of the existence of a hypercoagulable state triggered by the stress of the fast. In this respect, a few earlier reports^17–20^ have evaluated the effect of the RIF on hemostatic parameters. However, the most detailed of these studies by Kordy and Gader^17^ who found no significant differences in the blood levels of the coagulation factors: fibrinogen, FX, FV, FVII, FVIII, antithrombin (AT), and fibrinolytic parameters between measurements in Ramadan and ordinary non-fasting days. On the other hand, Sarraf-Zadegan et al.^18^ reported significantly decreased levels of fibrinogen and factor VII activity during RIF. Others^19^ found no change in PT, APTT, and fibrinogen levels, but D-dimer levels were significantly lower at the end of Ramadan than pre-and post-fasting levels^19^.

Our comprehensive understanding of the hemostatic changes during RIF will not be conclusive without information on natural inhibitors of blood coagulation (AT, proteins C and S). These earlier studies provided minimum information on antithrombotic components, particularly the natural anticoagulant protein S (PS).

Protein S is a vitamin K-dependent glycoprotein synthesized mainly by hepatocytes, macrophages, and endothelial cells^21^. It circulates in the plasma in two forms: free (40%), which expresses anticoagulant activity. In contrast, the rest (60%) is bound to C4 binding protein (C4BP) and has no anticoagulant activity^22,23^. Protein S functions as a cofactor that facilitates the inhibitory action of activated protein C on activated factor V (FVa) and activated factor VIII (FVIIIa). Besides, PS functions as a cofactor of tissue factor pathway inhibitor (TFPI), which inhibits the activity of tissue factor^24^, the main trigger of blood coagulation in vivo. Protein S deficiency is among the most frequent risk factors in thrombophilia^25^ and is clinically a risk factor for thromboembolic disease^26–28^.

Therefore, this case control study hypothesizes that the IF during Ramadan has the potential to alter the levels of the natural anticoagulants (AT, PC and PS) and operate as a protective antithrombotic mechanism. Thus, the current study aims to evaluate the effect of observing RIF on the blood levels of natural anticoagulants, antithrombin, protein C (PC), and total and free PS in healthy fasting people observing the religious IF of Ramadan.

## Materials and methods

This is a case–control study that included a total of 69 healthy Saudi participants who were randomly recruited from the staff of King Khalid University Hospital and healthy volunteers from other places (Table 1). The participants were divided into two groups. Group I is the fasting group and constitutes 29 healthy participants (mean age 31.2 ± 6.6 years, female: male ratio was 13:16) from which the blood samples were obtained in the afternoon after at least 8–12 h of complete fasting by the third week of Ramadan during a working day. Group II is the non-fasting group and consists of 40 non-fasting healthy volunteers who were all blood donors (mean age 30.5 ± 6.2 female: male ratio is 24:16) in whom blood samples were taken 2–4 weeks before the month of Ramadan after at least 8–12 h of complete fasting. Individuals with acute or chronic diseases or on medications during this study were excluded. Post-hoc power analysis was done using G*Power (version 3.1) from the obtained means ± SD of free PS comparing the difference between fasting and non-fasting state (n= 16 per group). The obtained effect size with an α = 0.05 was 0.91 and an achieved power of 0.70. The study protocol was approved by the Institutional Review Board of the College of Medicine, King Saud University (KSU), and written informed consent was obtained from all the participants who enrolled in the study. All methods were performed in accordance with the approved guidelines and complied with the Declaration of Helsinki.

**Table 1:** Demographic data of the study groups.

Parameter	Fasting group (n = 29)	Non-fasting group (n = 40)
Age (years)	31.2 ± 6.6	30.5 ± 6.2
Sex (Female:Male)	13:16	24:16
Body mass index (BMI, kg/m^2^)	25.8 ± 4.5	26.3 ± 6.4

### Blood collection and processing

The methods describing blood collection and processing have been published previously^29^. Ten ml of blood were collected by venipuncture, directly into vacutainer tubes containing 0.5 ml sodium citrate (3.8%, 0.129 mol/l; Terumo, Tokyo Japan), to give a ratio of nine volumes of blood to one volume of citrate. Proper mixing of blood with the anticoagulant was attained by gentle inversion. The usual precautions of selecting an easily accessible vein in the antecubital fossa and using the minimum venous stasis were observed. Blood samples were transported without delay to the Coagulation Research Laboratory, Physiology Department, College of Medicine/ King Khalid University Hospital. The blood tubes were centrifuged at 3000 r.p.m. (1000*g*), for 15 min, in a refrigerated (4–6 °C) centrifuge (model J-68; Beckman, New York, USA). Platelet-poor plasma was separated using plastic pipettes, and aliquots of plasma were immediately stored at minus 80 °C, until laboratory processing in batches, at a later date.

### Laboratory assays

Coagulation screening tests including PT, APTT, and fibrinogen were performed by the conventional methods (Diagnostica Stago, Asnières-sur-Seine, France), and the clotting times were registered by an optical coagulation system (ST-ART; Diagnostica Stago).

Coagulation inhibitors were assayed using an automated coagulometer (Stago START 4), and reagents were supplied by Diagnostica Stago, Asnières-sur-Seine, France and according to the manufacturer’s instructions: antithrombin by colorimetric assay (Stachrom Antithrombin Kit); protein C by colorimetric assay (STA-Stachrom PC) and total and free PS by enzyme-linked immunoassay (ELISA assay; Asserachrom total and free protein S). C4BP (Liatest C4b-BP) by latex immunoassay^30^.

### Statistical analysis

Data was analyzed using IBM SPSS version 21. Frequencies were presented as n and continuous variables were presented as mean ± standard deviation (SD). Kolmogorov–Smirnov and Shapiro–Wilk tests were used to test for normality. Independent Student’s T-test was done to compare fasting and non-fasting groups. Significance was set at p-value < 0.05.

## Results

The basic demographic data of the fasting participants (Table 1) did not show any significant differences in age and body mass index (BMI, kg/m^2^) between the study groups. The mean age of the fasting group was 31.2 ± 6.6 years, more than half (55.1%) of whom were males. The mean age of the participants in the non-fasting group was 30.5 ± 6.2, of whom 60% were females.

### Coagulation screening tests

**Figure 1:**
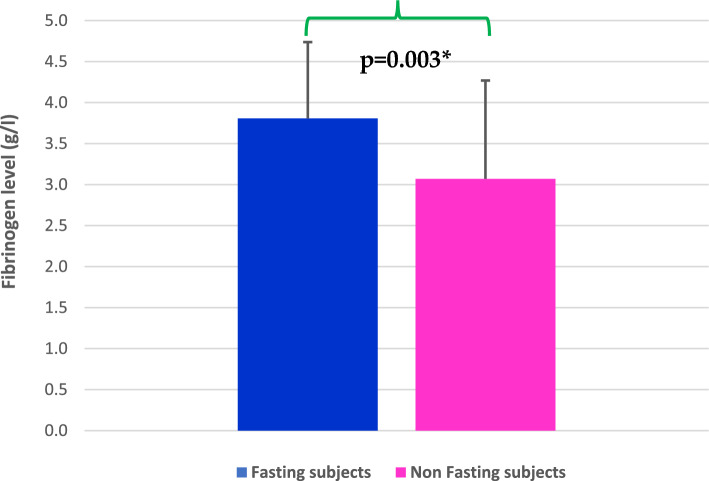
Plasma fibrinogen level in the fasting and non-fasting groups. The data are presented as (mean ± SD) in the different groups. *Indicates a significant difference at p < 0.05 as compared with the control group.

There was no significant difference in PT and APTT between fasting participants and the non-fasting group (Table 2). On the other hand, the mean fibrinogen level was higher in the fasting group (3.8 ± 0.9 g/l) in comparison with the non-fasting one (3.2 ± 0.8 g/l), (p < 0.05) (Fig. 1).

**Table 2:** Summary of the results of the coagulation screening tests in the study groups.

Parameters	PT (sec)	APTT (sec)	Fibrinogen (g/l)
Fasting group (n = 29)	14.2 ± 0.6	34.8 ± 1.9	3.8 ± 0.9*
Non-fasting group (Control) (n = 40)	14.2 ± 1.2	34.9 ± 1.5	3.2 ± 0.8
p value	0.80	0.80	0.003

### Natural coagulation inhibitors

#### Antithrombin (AT) and protein C (PC)

No significant differences were observed in the levels of the mean value of AT (103.7 ± 5.9, 101 ± 12.3% respectively) and PC (98.4 ± 17.2, 102.2 ± 18.7% respectively) in fasting participants as compared with the normal control group (Table 3).

**Table 3:** Summary of the results of the natural anticoagulants in the study groups.

Parameters	Antithrombin (%)	Protein C (%)	Protein S (Total) (%)	Protein S (Free) (%)	C4BP (%)
Fasting group (n = 29)	103.7 ± 5.9	98.4 ± 17.2	93.3 ± 14.8*	82.6 ± 11.4*	101.9 ± 10.4
Non-fasting group (controls) (n = 40)	101 ± 12.3	102.2 ± 18.7	85.1 ± 11.5	69.5 ± 9.2	101.5 ± 14.7
p value	0.24	0.40	0.02	p < 0.001	0.89

#### Total and free protein S (PS)

**Figure 2:**
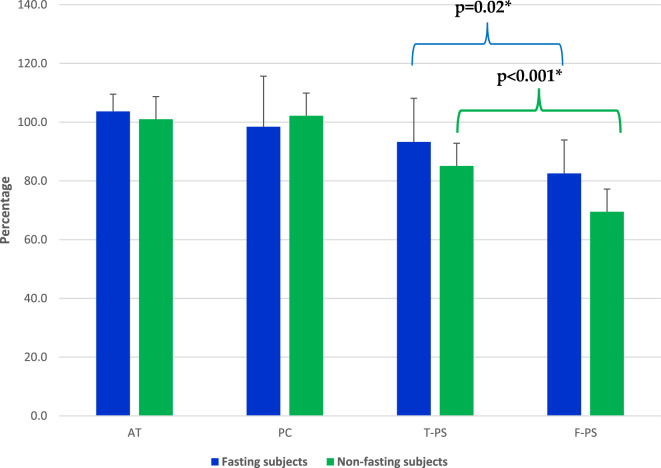
The Plasma level of the natural coagulation inhibitors; AT, PC and Total and Free PS in the fasting and non-fasting groups. The data are presented as (mean ± SD) in the studied groups. *Indicates a significant difference at p < 0.05 as compared with the control group.

It is of interest to note that the mean levels of both total and free PS were significantly higher in the fasting group than in the non-fasting group. That is, the total PS levels (93.3 ± 14.8%) were significantly higher in the fasting participants as compared with the normal controls (85.1 ± 11.5%, p < 0.05). The mean level of free PS in the fasting group was significantly higher (82.6 ± 11.4%) as compared with the non-fasting group (69.5 ± 9.2% p < 0.05) (Table 3 and Fig. 2).

#### C4 binding protein (C4BP)

No significant difference was observed in the mean level of C4BP between the fasting group (101.9 ± 10.4%) and the non-fasting group (101.5 ± 14.7%) (Table 3).

## Discussion

Several human and animal studies have shown that IF leads to health improvements in diabetes cardiovascular disease, obesity, cancers, and neurological disorders^31^. In this respect, the religious IF during the Muslim holy month of Ramadan has also been the subject of numerous biochemical^1–4^, hematological^5^, and hormonal^6,7^ studies that did not reflect serious disturbances in these measured parameters. On the other hand, other studies have reported a noticeable increase in the incidence of cerebrovascular thrombosis, retinal vein occlusion, sinus venous thrombosis^10–13^, and acute coronary syndrome^14,15^ during Ramadan. This raises the question of whether disturbances in the hemostatic system might contribute to the increased susceptibility to the development of cardiovascular and cerebrovascular thrombosis during Ramadan fasting month.

Some earlier hemostatic studies did not confirm the presence of prothrombotic hemostatic blood changes during Ramadan fasting. One report found significantly decreased levels of fibrinogen and factor VII activity during Ramadan fasting^18^. Besides, a previous detailed study from our institution reported no significant differences in the blood levels of the coagulation factors; fibrinogen, FX, FV, FVII, FVIII, antithrombin, and fibrinolytic parameters between Ramadan and ordinary non-fasting days^17^. Similarly, Others^19^ found no change in PT, APTT, and fibrinogen levels and significantly lower D-dimer levels at the end of Ramadan in comparison to pre/post-fasting levels^19^. To explore the pathophysiology of increased tendency of thrombosis during Ramadan fasting, we evaluated the effect of Ramadan fasting on the natural anticoagulants, AT, PC, and PS levels in healthy participants after 20 days of IF. These natural anticoagulants, particularly PS, did not seem to have caught the interest of previous researchers.

The natural anticoagulants AT, PC, and PS play essential roles in the regulation of the coagulation pathway as they limit excess fibrin formation at the site of endothelial injury, thus reducing the risk of thrombus formation. Therefore, it is not surprising that congenital or acquired deficiency of these natural anticoagulants will result in an increased risk of thrombosis^32^. The importance of the anticoagulant role of PS is evident from the consequences of its inherited deficiency, which is associated with an increased risk of venous thromboembolism and fetal loss. Individuals with heterozygous PS deficiency, most commonly present with deep venous thrombosis, pulmonary embolism, and superficial thrombophlebitis^33^.

Interestingly, in the current study, we found that RIF was associated with increased levels of both total and free PS but no significant variations in AT and PC levels. A careful search of the literature failed to find any reports on PS levels in fasting healthy participants. Schwarz et al. (1988) reported high levels of free PS in newborn infants which were associated with low levels of total PS and C4BP^34^. These authors proposed that the increased protein S activity might be a physiological protective mechanism against thrombosis in these infants^34^. In another family study, Comp et al. (1990) reported increased free protein S level, which was attributed to a hereditary deficiency of the carrier protein, C4BP. This elevation of free protein S was not associated with abnormal hemostasis, such as increased hemorrhagic tendency^35^. Similarly, another case study of a 34-year-old female with ischemic retinopathy found increased free protein S with C4BP deficiency. The authors attributed the increased risk of arterial thrombosis in this patient to the low PS/C4BP complex level^36^. High total protein S concentrations were also reported in patients with a history of angina pectoris and myocardial infarction^37^. This was confirmed further by Ken-Dror et al. (2011), who reported an association between high free protein S levels and coronary heart disease (CHD) risk over a 7-year follow-up^38^. The pathogenic significance of increased protein S levels in men at high risk of CHD and stroke was unclear. However, the increase in protein S levels was attributed to the heightened, inappropriate, immune response as a result of atherosclerosis in CHD^38^.

In the present study, the mechanism responsible for the elevated levels of free PS in fasting participants is open to speculation. It is most probably due to either increased synthesis of PS or a change in the distribution between free PS and PS complexed to C4bBP. In contrast to the above-mentioned three studies^34–36^, which related the increased free protein S levels to the decrease in the levels of the carrier protein C4BP, in the current study, there was no difference in C4bBP levels between fasting participants and the non-fasting group. This suggests that the increase in PS production by endothelial cells is mostly the main reason for the rise in PS levels. PS is synthesized and secreted mainly by hepatocytes, macrophages^39^ and endothelial cells^40^. The endothelial cells production of protein S is down-regulated by the proinflammatory cytokine tumor necrosis factor-α (TNF-α)^41^. Low-calorie intake in animal models has been shown to affect the production of proinflammatory cytokines and reduce their levels^42^. Faris et al. (2012) measured the proinflammatory cytokines IL-1β, IL-8, IL-6, and TNF α at the end of the third week of Ramadan and found significantly low plasma levels of these cytokines as compared to levels before or after RIF^43^. Therefore, these findings gave strong support to the speculation that during the fasting period in Ramadan, the reduction in the endothelial cells production of cytokines, particularly TNF-α and IL-6, will dimmish their downregulation effect on endothelial cell synthesis of PS, resulting in increased production of PS. Ultimately a high PS level might act as an ideal physiological mechanism to protect against the risk of thrombosis or clot formation in healthy fasting people during Ramadan. This effect will complement the known beneficial effects of IF in promoting cardiometabolic protective effect^8,9^ and in reducing the elevated inflammatory (CRP, IL-1, IL-6, TNF-α) and oxidative stress markers, malondialdehyde^44^.

Recently, the health effects of IF have been ascribed to the effect of ketone bodies. The lack of food during fasting leads to the preferential breakdown of fat, with a major contribution of ketone bodies serving as fuel. Many beneficial non-metabolic actions of fasting, such as increased anti-oxidative and anti-inflammatory activity, cell repair, and regeneration mechanisms, have been attributed to the effect of ketone bodies on organ functions^45^. Currently, there are no studies on ketone bodies together with hemostatic markers during Ramadan fasting. Thus, future research is needed to determine the role of ketone bodies in protection against thrombosis.

Plasma fibrinogen concentration is becoming a widely recognized risk factor for cardiovascular and cerebrovascular diseases^46–48^. However, fasting during Ramadan does not have any significant influence on fibrinogen levels. In the present study, we observed a small increase in fibrinogen levels in fasting participants as compared to non-fasting ones. While decreased fibrinogen levels have been reported during Ramadan fasting^18^, others^17,19^ found no difference in fibrinogen levels between Ramadan and ordinary non-fasting days. We can only draw from these observations that Ramadan fasting has no significant effect on the kinetics of the acute phase protein, fibrinogen.

The question of whether the stress of the RIF would induce a hypercoagulable prothrombotic state resembling that associated with other forms of stress^49^ is a matter of debate. When observing the fasting, Muslims are also obliged to perform many religious rituals which make peace of mind a salient feature of Ramadan. As a consequence, RIF puts much less mental strain on daily life than what is normally encountered on ordinary non-fasting days. This is supported by several recent studies that showed the positive effects of fasting on mental health, such as reducing stress, anxiety, depressive symptoms, and insomnia. Fasting and the spiritual and social practices that accompany it enable fasting individuals to reach a state of tranquillity^50–52^.

The present study highlights unique information on the physiology of hemostasis during RIF. This was the first study to evaluate the levels of coagulation inhibitors during Ramadan fasting. The importance of our findings of elevated levels of the coagulation inhibitors, total and free PS, in addition to the presumed reduction of stress associated with Ramadan fasting, offer a potential explanation for the absence of thrombosis in fasting healthy people which operate as physiological mechanisms that protect against thrombosis development. These novel findings are particularly significant because they expand our understanding of the physiological complex mechanisms behind the health-improving effects of IF, in general, and particularly during Ramadan.

This study has several limitations. The major limitation of this study is the small sample size. Also, the design of the study, which was based on the assessment of the variables at a single time point, precluded the serial changes in the levels and activity of the natural anticoagulants. Furthermore, additional markers of hypercoagulation or inflammation were not included in this study.

## Conclusion

Our data showed that the RIF is associated with changes in the dynamic of natural coagulation inhibitors, particularly PS. Elevated levels of total and free PS with no change in C4BP level might provide the fasting people with a vital physiological anticoagulant mechanism that may be associated with temporal protection against the risk of the development of thrombosis in healthy fasting people.

The above findings highlight the need for more detailed hemostatic studies with a larger sample size to confirm this association between fasting and the changes in the levels of natural anticoagulants in healthy and nonhealthy participants during Ramadan fasting.

## Data Availability

The data presented in this study are available on reasonable request from the corresponding author.
